# Diagnostic performance of endocytoscopy with normal pit‐like structure sign for colorectal low‐grade adenoma compared with conventional modalities

**DOI:** 10.1002/deo2.238

**Published:** 2023-05-08

**Authors:** Kenichi Suzuki, Shin‐ei Kudo, Toyoki Kudo, Masashi Misawa, Yuichi Mori, Katsuro Ichimasa, Yasuharu Maeda, Takemasa Hayashi, Kunihiko Wakamura, Toshiyuki Baba, Fumio Ishda, Shigeharu Hamatani, Haruhiro Inoue, Kazunori Yokoyama, Hideyuki Miyachi

**Affiliations:** ^1^ Digestive Disease Center Showa University Northern Yokohama Hospital Kanagawa Japan; ^2^ Suzuki Gastrointestinal Clinic Akita Japan; ^3^ Tokyo Endoscopy Clinic Tokyo Japan; ^4^ Clinical Effectiveness Research Group Institute of Health and Society University of Oslo Oslo Norway; ^5^ Hamatani‐kikaku Tokyo Japan; ^6^ Digestive Disease Center Showa University Koto Toyosu Hospital Tokyo Japan; ^7^ Department of Gastroenterology Nikko Memorial Hospital Hokkaido Japan

**Keywords:** colonoscopy, low grade adenoma, endocytoscopy, resect and discard, normal pit (NP) sign

## Abstract

**Objectives:**

A “resect‐and‐discard” strategy has been proposed for diminutive adenomas in the colorectum. However, this strategy is sometimes difficult to implement because of the lack of confidence in differentiating low‐grade adenoma (LGA) from advanced lesions such as high‐grade adenoma or carcinoma. To perform real‐time precise diagnosis of LGA with high confidence, we assessed whether endocytoscopy (EC) diagnosis, considering normal pit‐like structure (NP‐sign), an excellent indicator of LGA, could have additional diagnostic potential compared with conventional modalities.

**Methods:**

All the neoplastic lesions that were observed by non‐magnifying narrow‐band imaging (NBI), magnifying NBI (M‐NBI), magnifying pit pattern, and EC prior to pathological examination between 2005 and 2018 were retrospectively investigated. The neoplastic lesions were classified into two categories: LGA and other neoplastic lesions. We assessed the differential diagnostic ability of EC with NP‐sign between LGA and other neoplastic lesions compared with that of NBI, M‐NBI, pit pattern, and conventional EC in terms of sensitivity, specificity, accuracy, and area under the receiver operating characteristic curve (AUC).

**Results:**

A total of 1376 lesions from 1097 patients were eligible. The specificity (94.9%), accuracy (91.5%), and area under the receiver operating characteristic curve (0.95) of EC with NP‐sign were significantly higher than those of NBI, M‐NBI, pit pattern, and conventional EC.

**Conclusions:**

EC diagnosis with NP‐sign has significantly higher diagnostic performance for predicting colorectal LGA compared with the conventional modalities and enables stratification of neoplastic lesions for “resect‐and‐discard” with higher confidence.

## INTRODUCTION

In recent years, the widespread application of cold polypectomy has made it safer to resect colorectal polyps ≤5 mm in size. Most diminutive polyps are benign adenomas^1^ and have an extremely low prevalence of invasive cancer and advanced histology.^2^ Consequently, a “resect‐and‐discard” strategy has been proposed for diminutive adenomas, which reduces the time, effort, and cost of collecting specimens and performing histopathological diagnosis.^3^, ^4^, ^5^ However, some colorectal cancers ≤5 mm in size have been confirmed.^6^ Furthermore, small colorectal carcinomas with no evidence of any adenomatous remnant, which may have followed a “de novo” pathway, have recently been reported.^7^, ^8^ Therefore, real‐time precise endoscopic diagnosis with high confidence is a prerequisite for the “resect‐and‐discard” strategy using cold snare polypectomy.

Magnifying narrow‐band imaging (M‐NBI) and magnifying pit pattern (PIT) diagnosis are used for the differential diagnosis of colorectal neoplastic lesions. M‐NBI and PIT diagnosis provides additional diagnostic value for predicting neoplastic lesions compared with conventional diagnosis.^9^, ^10^ However, there are some colorectal lesions with low confidence even when using these modalities in our routine colonoscopy. The diagnostic accuracies of colorectal polyps initially diagnosed with low confidence are usually low with M‐NBI (58.5%) and with PIT (66.0%), respectively.^11^

Endocytoscopy (EC) can magnify endoscopic images to a maximum magnification of 520 times, thereby facilitating real‐time histopathologic endoscopic diagnosis.^12^, ^13^ EC has been successfully used for examination of the esophagus,^12^ stomach,^14^ duodenum,^15^ and colon.^13^ For colorectal lesions, EC has been used to differentiate between neoplastic and non‐neoplastic lesions and between massively invasive submucosal cancers and other neoplastic lesions,^16^ regardless of lesion size. It is currently often difficult to confidently distinguish between LGA lesions that can be “resected‐and‐discarded” from high‐grade adenomas/villous adenoma lesions that should be pathologically diagnosed after resection. Nevertheless, we previously demonstrated that the normal pit‐like structure (NP‐sign) observed in EC was an excellent indicator of colorectal low‐grade adenoma (LGA).^17^

To date, no study has examined whether EC diagnosis with NP‐sign has the additional diagnostic ability for LGA compared with conventional modalities. In this retrospective analysis, we examined the differential diagnostic ability of EC diagnosis with NP‐sign for colorectal LGA compared with that of NBI, M‐NBI, PIT, and conventional EC diagnosis.

## METHODS

### Study design and clinical data

This study was a single‐center, retrospective study that was conducted in the Digestive Disease Center at Showa University Northern Yokohama Hospital. The study included colorectal neoplastic lesions that were observed by NBI, M‐NBI, PIT, and EC prior to endoscopic or surgical resection between January–May 2005 and May 2018. We excluded serrated lesions including hyperplastic polyps and sessile serrated lesions. Non‐neoplastic lesions such as inflammatory polyps and juvenile polyps, non‐epithelial lesions such as malignant lymphoma, lesions associated with ulcerative colitis, and poor‐image lesions that could not withstand evaluation were also excluded. On the basis of histopathology, we divided the neoplastic lesions into two groups: LGA and advanced lesions, which we defined as high‐grade adenoma, villous adenoma, and invasive carcinoma.^17^ As the main outcome, we determined the differential diagnostic performance of EC diagnosis with NP‐sign for colorectal LGA compared with that of NBI, M‐NBI, PIT, and conventional EC diagnosis in terms of the sensitivity, specificity, positive predictive value (PPV), negative predictive value (NPV), accuracy and area under the receiver operating characteristic curve (AUC). As a secondary outcome, the diagnostic performance of lesions less than 5 mm was also compared in terms of sensitivity, specificity, PPV, NPV, and accuracy. Data on patient age, sex, tumor size, and tumor location were reviewed from the electronic record system. Tumor morphology was classified following Kudo's morphological development classification.^18^

### Ethical approval

The study protocol was approved by the Ethics Committee of Showa University Northern Yokohama Hospital (no.19H039; December 9, 2019) and was registered as a University Hospital Medical Information Network clinical trial (UMIN000038117). This study was performed in accordance with the Declaration of Helsinki.

### Endoscopic procedures

Bowel preparation involved the administration of 2.0–3.0 L of polyethylene glycol solution in the morning before the procedure. Colonoscopy was performed using a video endoscopic system (EVIS, Lucera Spectrum; Olympus Optical Co. Ltd., Tokyo, Japan) with an integrated‐type endocytoscope (CF Y‐0001 and CF Y‐0020‐I; prototypes from Olympus) and an endocytoscope (CF‐H290ECI; Olympus) with a maximum magnification of ×520. Non‐magnifying NBI diagnosis was performed following the NBI International Colorectal Endoscopic classification.^19^ M‐NBI diagnosis was performed using the Japanese NBI Expert Team classification.^19^ PIT diagnosis was performed by staining with 0.4% indigo carmine dye, which was replaced with 0.05% crystal violet staining as necessary, following colorectal pit pattern classification.^20^ We then conducted double‐staining with 0.05% crystal violet and 1% methylene blue^21^ and performed the EC diagnosis following the EC classification.^16^ Endoscopic observation was performed in the order of NBI, M‐NBI, PIT, and EC, and the diagnosis was determined after each observation. We used the real‐time diagnosis of endoscopic findings in NBI, M‐NBI, PIT, and EC by the endoscopist at the time of examination and did not correct the findings by later reviewing the images.

### NP‐sign

**FIGURE 1:**
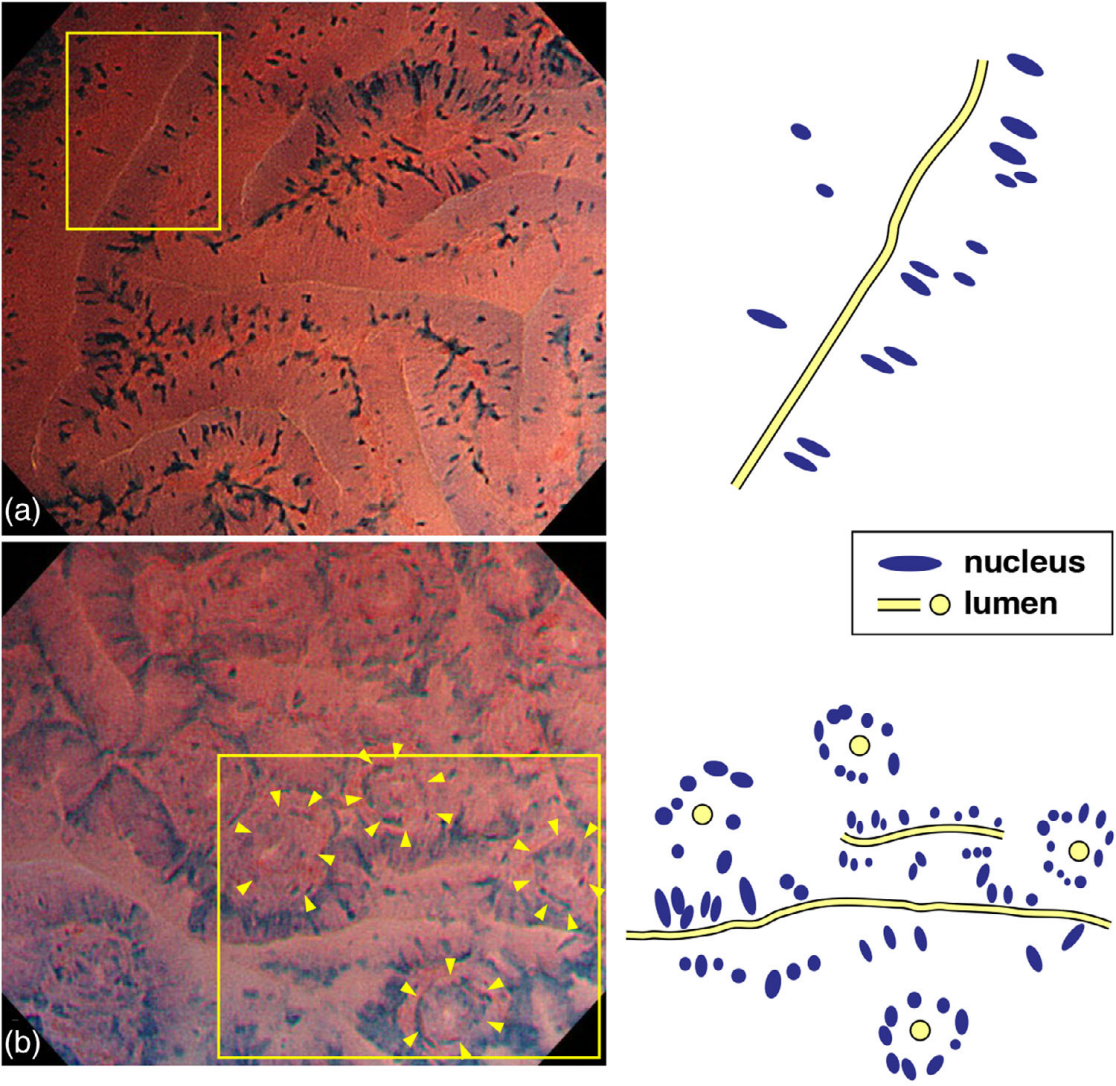
Normal pit–like structure (NP‐sign). (a) NP‐sign negative lesion. The lesion has smooth, clear slit‐like lumens and a regular pattern of fusiform or roundish nuclei in endocytoscopic image. The nuclei are slightly swollen compared with nuclei in normal mucosa. (b) NP‐sign positive lesion. Small round lumens such as a normal pit are interposed in a smooth slit lumen. When the NP was observed in at least one field of view at maximum magnification, the lesion was defined as NP‐sign positive. The arrow tip indicates the nuclei, which form a small round lumen like a normal pit.

**FIGURE 2:**
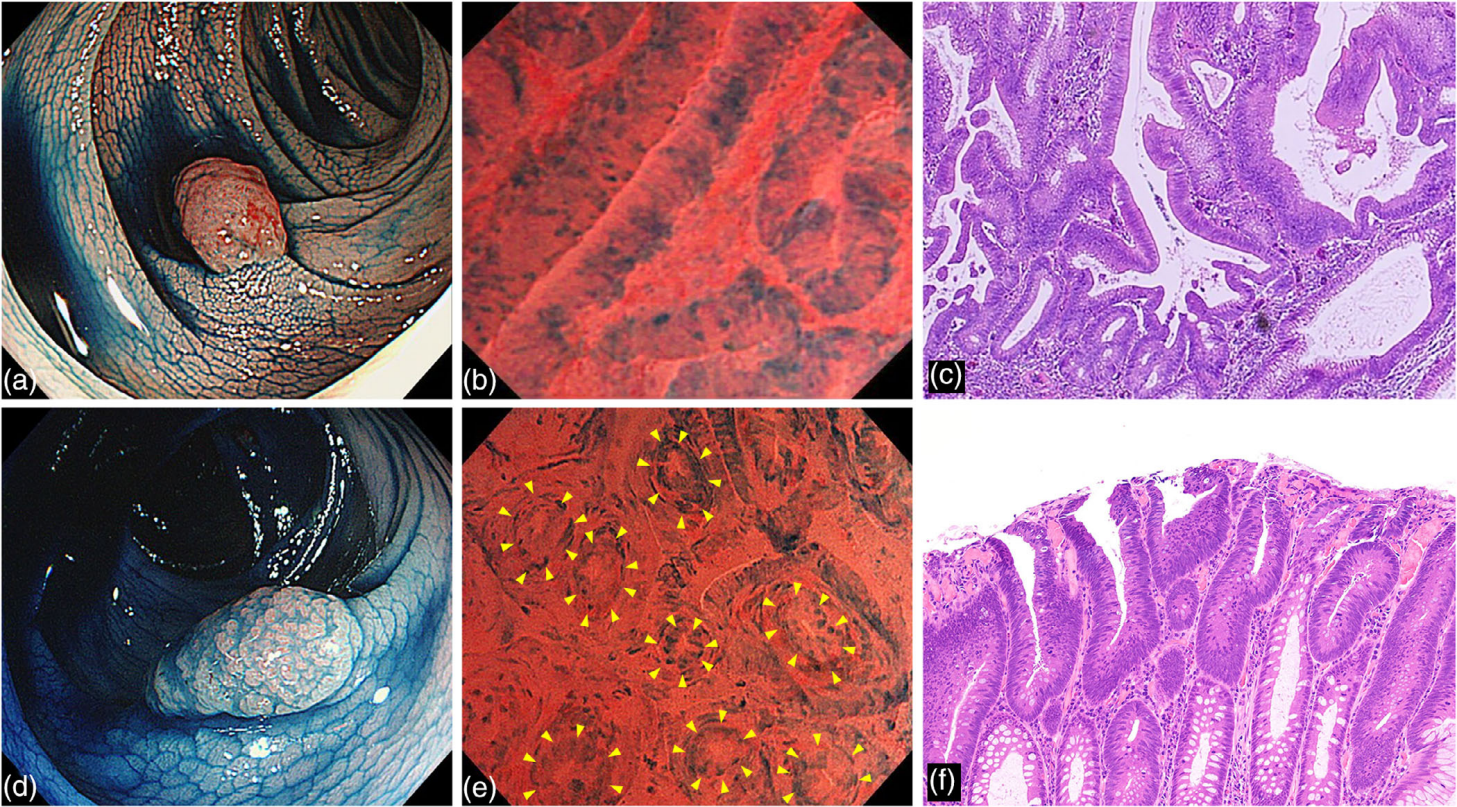
NP‐sign negative lesion and –positive lesion. (Top panel) An NP‐sign negative lesion: (a) White‐light image with indigo carmine dye staining, showing a 0–I s lesion in the sigmoid colon that was approximately 8 mm in size. (b) In the endoscopic image, a round lumen is not observed; only smooth slit‐like lumens were detected. This lesion is diagnosed as NP‐sign negative. (c) Pathological image of the lesion showing high‐grade adenoma (intra‐mucosal carcinoma) in tubular adenoma. (Bottom panel) An NP‐sign positive lesion: (d) White‐light image with indigo carmine dye staining, showing a 0–I s lesion of the transverse colon that is approximately 8 mm in size. (e) Endocytoscopic image showing multiple round lumens like type I pit interposed in a smooth slit lumen. The arrow tip indicates the nuclei, which form a small round lumen like a normal pit. (f) Pathological image of the lesion, showing a low‐grade tubular adenoma. NP‐sign, normal pit–like structure sign.

In the EC classification, EC 2 is composed of slit‐like lumens and regularly arranged fusiform or roundish nuclei, representing adenomatous lesions. Some studies, however, reported that invasive cancers were included among EC 2 lesions,^17^, ^22^ suggesting the risk of discarding invasive cancers. Among EC 2 lesions, some lesions have an NP‐sign side by side with slit‐like lumens, and this EC finding was defined as NP‐sign in our previous study.^17^ Lesions in which the NP was not observed were classified as NP‐sign negative (Fig. 1). In this study, all neoplastic lesions observed by EC were divided into two groups: NP‐sign positive lesions and NP‐sign negative lesions. If there was at least one normal pit‐like roundish lumen in one field of ultra‐high magnification, the lesion was classified as NP‐sign positive. The presence of NP‐sign in EC images was decided by the first author (Kenichi Suzuki) and the third author (Toyoki Kudo), who reviewed all the EC images of the lesions. We did not review the pathological findings before evaluating NP‐sign. Representative NP‐sign positive and negative lesions are shown in Figures 1 and 2.

### Endoscopic findings as factors indicating LGA

NBI, M‐NBI, PIT, and EC diagnosis corresponding to histopathological images of LGA are as follows; NBI diagnosis is type 2,^19^ M‐NBI diagnosis is type 2A,^19^ PIT diagnosis is type III_L_,^20^ and EC diagnosis is EC 2.^16^ In addition to the diagnosis by conventional EC classification, EC with NP‐sign was investigated as an indicator of LGA.

### Pathological evaluation

The specimens were examined by one pathologist using the World Health Organization criteria^23^ and the Japanese Society for Cancer of the Colon and Rectum guidelines,^24^ as previously described.^25^

### Statistical analyses

To evaluate the diagnostic ability of LGA of NBI, M‐NBI, PIT, conventional EC, and NP‐sign EC, McNemar's test was used to evaluate the sensitivity, specificity, and accuracy, and the chi‐square test with weighted generalized statistics was used to evaluate the PPV and NPV. The chi‐square test was used to evaluate the diagnostic ability of LGA in each modality and receiver operating characteristic curve. Data are presented as the mean ± standard deviation. A two‐sided *p*‐value < 0.05 was considered statistically significant. Statistical analyses were performed using SPSS version 22 (IBM Corp., Armonk, NY, USA) and R version 3.6.3 (R Foundation for Statistical Computing, Vienna, Austria).

## RESULTS

### Clinicopathological features of the patients and lesions

**FIGURE 3:**
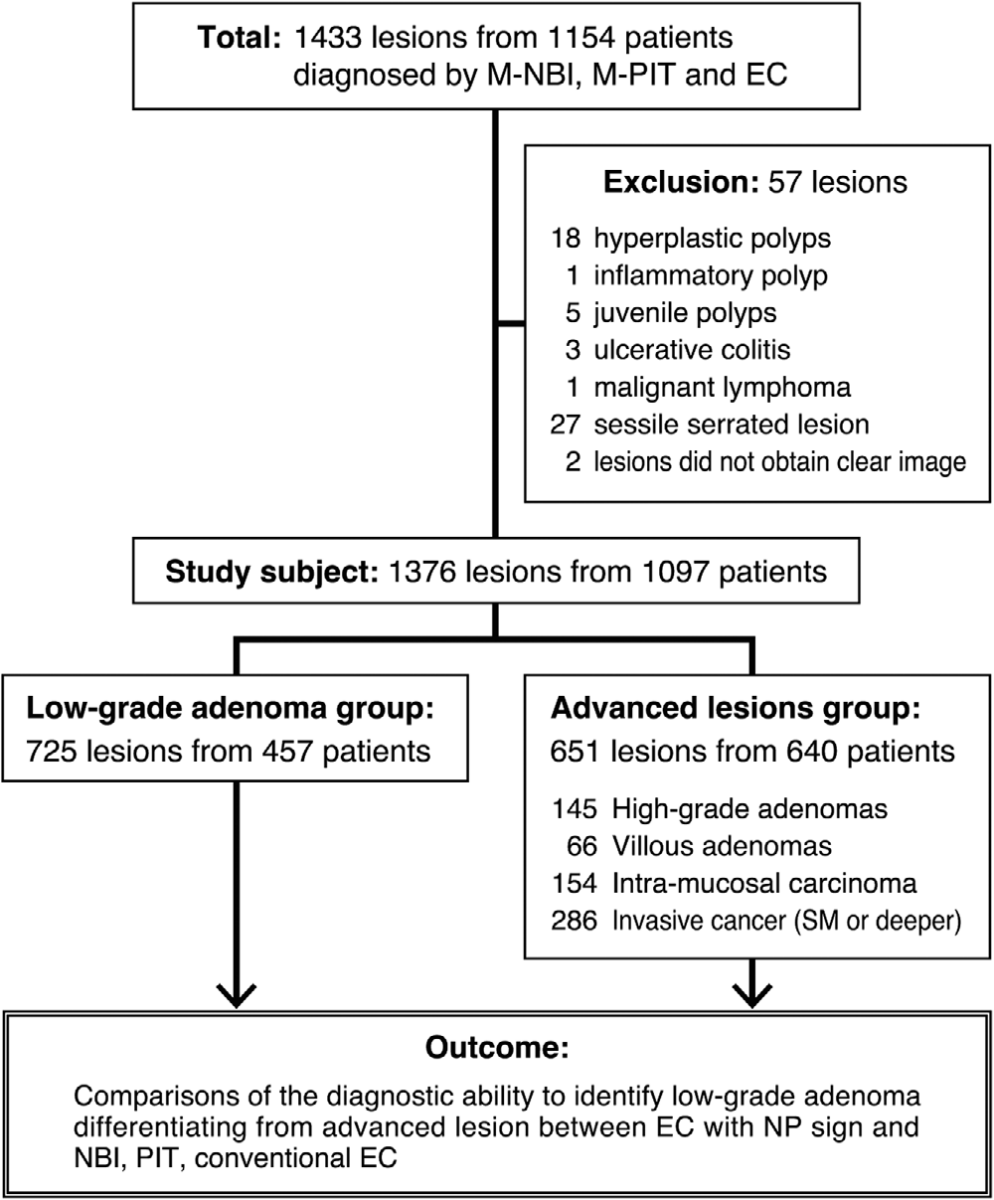
Study flow chart. Colorectal neoplastic lesions that were observed by NBI, M‐NBI, PIT, and EC prior to endoscopic or surgical resection were retrospectively examined. Serrated and non‐neoplastic and non‐epithelial lesions such as a hyperplastic polyp, sessile serrated lesion, inflammatory polyp, and malignant lymphoma were excluded. Lesions associated with ulcerative colitis and poor‐image lesions that could not withstand evaluation were also excluded. From the histopathology, we divided all the neoplastic lesions into two groups: LGA and advanced lesion, which we further defined high‐grade adenoma, villous adenoma, and invasive carcinoma. Finally, we elucidated the differential diagnostic performance of EC diagnosis with NP‐sign for colorectal LGA compared with that of NBI, M‐NBI, PIT, and conventional EC diagnosis. EC, endocytoscopy; NP‐sign, normal pit–like structure sign; NBI, narrow‐band imaging; M‐NBI, magnifying narrow‐band imaging; PIT, magnifying pit pattern; LGA, low‐grade adenoma.

A flow chart of the study analysis is shown in Figure 3. A total of 1433 colorectal lesions from 1154 patients (831 men and 323 women) were observed by NBI, M‐NBI, PIT, and EC prior to endoscopic or surgical resection. We excluded 57 lesions: 18 hyperplastic polyps, 27 sessile serrated adenoma/polyps, one inflammatory polyp, five juvenile polyps, one malignant lymphoma, three lesions associated with ulcerative colitis, and two poor‐image lesions. Finally, 1376 lesions from 1097 patients (791 men and 306 women) were assessed in this study. Among these lesions, 506 lesions were smaller than 5 mm.

The background clinicopathological features of the patients and lesions are listed in Table 1. Regarding morphological types, 544 lesions (39.5%) were protruded‐type, 621 lesions (45.1%) were flat‐type, 114 lesions (8.3%) were depressed‐type, and 97 (7.1%) lesions were advanced cancers (T2 or deeper). The histopathological evaluation confirmed 725 LGAs (52.7%), 299 high‐grade adenomas (21.7%), 66 villous adenomas (4.8%), and 286 invasive carcinomas (20.8%).

**TABLE 1:** Clinicopathological features of the patients and lesions.

Number of patients/lesions	1097/1376
Sex	
Male	782
Female	315
Mean age (years old)	66.5 ± 10.6
Mean size of lesions (mm)	12.2 ± 11.8
Location (%)	
Cecum	92 (6.7%)
Ascending colon	277 (20.1%)
Transverse colon	266 (19.3%)
Descending colon	115 (8.4%)
Sigmoid colon	383 (27.8%)
Rectum	243 (17.7%)
Morphological types (%)	
Protruding (0‐Ip, 0‐Is, 0‐ Isp)	544 (39.5%)
Flat (0‐IIa)	621 (45.1%)
Depressed (0‐IIc, 0‐IIa+IIc, 0‐IIc+IIa, 0‐Is+IIc)	114 (8.3%)
Advanced‐type cancers (T2 and above)	97 (7.1%)
Histopathology of resected specimens (%)	
LGA	725 (52.7%)
HGA	299 (21.7%)
VA	66 (4.8%)
Carcinoma with submucosal invasion	156 (11.4%)
Carcinoma with muscularis propria invasion	76 (5.5%)
Carcinoma with subserosal invasion	54 (3.9%)
Treatment (%)	
Polypectomy	514 (37.4%)
EMR	556 (40.4%)
ESD	58 (4.2%)
Surgical resection	248 (18.0%)

Abbreviations: EMR, endoscopic mucosal resection; ESD, endoscopic submucosal dissection; HGA, high‐grade adenoma; LGA, low‐grade adenoma; VA, villous adenoma.

### Comparisons of the diagnostic ability for LGA

Table 2 shows the correlations between the histopathological findings and endoscopic diagnoses by NBI, M‐NBI, PIT, and EC (with and without NP‐sign). None of the lesions were diagnosed as EC1. Table 3 shows the comparison of the diagnostic ability to identify LGA between EC with NP‐sign and the four modalities. The specificity, PPV, and accuracy of EC with NP‐sign were significantly higher than those with NBI, M‐NBI, PIT, and conventional EC (*p* < 0.001). The sensitivity of EC with NP‐sign was significantly higher than that of NBI (*p* = 0.0315), but not significantly different from that of M‐NBI (*p* = 0.7453) and PIT (*p* = 0.896); it was significantly lower than that of conventional EC (*p* < 0.001). The NPV of EC with NP‐sign was significantly higher than that of NBI (*p* < 0.001) and M‐NBI (*p* = 0.0057), but not significantly different from PIT (*p* = 0.669); it was significantly lower than conventional EC (*p* < 0.001). Table 4 shows a comparison of the diagnostic ability to identify LGA in lesions smaller than 5 mm. The sensitivity, PPV, NPV, and accuracy of EC with NP‐sign were significantly higher than those of NBI (*p* < 0.001). There were no significant differences in sensitivity, specificity, PPV, NPV, and accuracy in comparison with M‐NBI, PIT, and EC diagnosis.

**TABLE 2:** Correlations between endoscopic diagnoses and histopathological findings.

*n* = 1376		LGA	HGA	VA	SM	MP	SS
NBI	1	112	32	7	1		
	2	613	198	59	38	28	2
	3		69		117	48	52
M‐NBI	1	20	19	3	1		
	2A	637	169	62	8	1	
	2B	68	103	1	81	28	1
	3		8		66	47	53
PIT	II	10	22	5	1		
	III	643	76	11	2		
	IV	64	120	48	8	1	
	V_I_	8	80	2	60	28	6
	V_N_		1		85	47	48
EC	2 NP‐sign (+)	641	24	2			
	2 NP‐sign (‐)	79	189	64	9	1	4
	3a	5	69		42	7	
	3b		17		105	68	50

Abbreviations: EC, endocytoscopy; HGA, high‐grade adenoma; LGA, low‐grade adenoma; M‐NBI, magnifying narrow‐band imaging; MP, carcinoma with muscularis propria invasion; NBI, narrow‐band imaging; NP‐sign, normal pit‐like structure sign; PIT, magnifying pit pattern; SM, carcinoma with submucosal invasion; SS, carcinoma with subserosal invasion; VA, villous adenoma.

**TABLE 3:** Comparisons of the diagnostic ability to identify low‐grade adenoma differentiating from advanced lesions^*^ between endocytoscopy (EC) with normal pit‐like structure sign (NP‐sign) and the other four modalities.

	Sensitivity (95% CI)	Specificity (95% CI)	PPV (95% CI)	NPV (95% CI)	Accuracy (95% CI)
EC with NP‐sign	88.4 (86.7–90.1)	94.9 (93.7–96.1)	95.1 (94.0–96.2)	88.0 (86.3–89.7)	91.5 (90.0–93.0)
NBI	84.6 (82.7–86.5)	50.1 (47.5–52.7)	65.4 (62.9–67.9)	74.4 (72.1–76.7)	68.2 (65.7–70.7)
*p‐*Value	0.032	<0.001	<0.001	<0.001	<0.001
M‐NBI	87.9 (86.2–89.6)	59.6 (57.0–62.2)	70.8 (68.4–73.2)	81.5 (79.5–83.6)	74.5 (72.2–76.8)
*p‐*Value	0.745	<0.001	<0.001	0.005	<0.001
PIT	88.7 (87.0–90.4)	83.4 (81.4–85.4)	85.6 (83.7–87.5)	86.9 (85.1–88.7)	86.2 (84.4–88.0)
*p‐*Value	0.896	<0.001	<0.001	0.669	<0.001
EC	98.5 (97.9–99.1)	55.0 (52.4–57.6)	70.9 (68.5–73.3)	97.0 (96.1–97.9)	77.9 (75.7–80.1)
*p‐*Value	<0.001	<0.001	<0.001	<0.001	<0.001

McNemar's test was used for the sensitivity, specificity, and accuracy, and the chi‐square test with weighted generalized statistics was used for the PPV, NPV, and *p‐*values.

Abbreviations: CI, confidence interval; EC, endocytoscopy; LGA, low‐grade adenoma; M‐NBI, magnifying narrow‐band imaging; NBI, narrow‐band imaging; NP‐sign, normal pit‐like structure sign; NPV, negative predictive value; PIT, magnifying pit pattern; PPV, positive predictive value.

* Advanced lesions are composed of high‐grade adenoma, villous adenoma, and invasive carcinoma.

**TABLE 4:** Comparisons of the diagnostic ability to identify low‐grade adenoma differentiating from advanced lesions^*^ between endocytoscopy (EC) with normal pit‐like structure sign (NP‐sign) and the other four modalities in lesions less than 5 mm.

	Sensitivity (95% CI)	Specificity (95% CI)	PPV (95% CI)	NPV (95% CI)	Accuracy (95% CI)
EC with NP‐sign	98.6 (97.0–99.4)	59.1 (36.4–79.3)	98.1 (96.5–99.1)	65.0 (40.8–84.6)	96.8 (94.9–98.2)
NBI	89.1 (85.9–91.7)	9.1 (1.1–29.2)	95.6 (93.2–97.3)	3.6 (0.4–12.5)	85.6 (82.2–88.5)
*p‐*Value	<0.001	0.003	<0.001	<0.001	<0.001
M‐NBI	97.7 (96.0–98.9)	27.3 (10.7–50.2)	96.7 (94.7–98.1)	35.3 (14.2–61.7)	94.7 (92.3–96.5)
*p‐*Value	0.386	0.070	0.032	0.023	0.037
PIT	96.5 (94.4–97.9)	45.5 (24.4–67.8)	97.5 (95.7–98.7)	37.0 (19.4–57.6)	94.3 (91.9–96.1)
*p‐*Value	0.016	0.450	0.223	0.004	0.009
EC	99.2 (97.9–99.8)	40.9 (20.7–63.6)	97.4 (95.5–98.6)	69.2 (38.6–90.9)	96.6 (94.7–98.0)
*p‐*Value	0.546	0.343	0.214	0.781	1.000

McNemar's test was used for the sensitivity, specificity, and accuracy, and the chi‐square test with weighted generalized statistics was used for the PPV, NPV, and *p‐*values.

Abbreviations: CI, confidence interval; EC, endocytoscopy; LGA, low‐grade adenoma; M‐NBI, magnifying narrow‐band imaging; NBI, narrow‐band imaging; NP‐sign, normal pit‐like structure sign; NPV, negative predictive value; PIT, magnifying pit pattern; PPV, positive predictive value.

* Advanced lesions are composed of high‐grade adenoma, villous adenoma, and invasive carcinoma.

**FIGURE 4:**
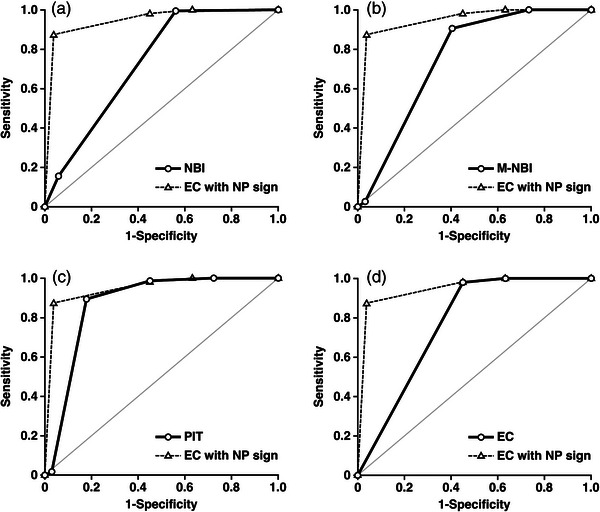
Comparisons using the receiver operating characteristic curve of the diagnostic ability to identify LGA between EC with NP‐sign and the other four modalities. Regarding the diagnostic ability to identify LGA, the area under the concentration‐time curve of EC with NP‐sign was significantly higher than that of NBI, M‐NBI, PIT, and conventional EC (*p* < 0.001). ROC, receiver operating characteristic curve; LGA, low‐grade adenoma; EC, endocytoscopy; NP‐sign, normal pit–like structure sign; AUC, area under the concentration‐time curve; NBI, narrow‐band imaging; M‐NBI, magnifying narrow‐band imaging; PIT, magnifying pit pattern.

Figure 4 shows the comparison using the receiver operating characteristic curve among the five modalities. The AUC was 0.95 (95% confidence interval: 0.94–0.96) for EC with NP‐sign; it was significantly higher than that of NBI, M‐NBI, PIT, and conventional EC (*p* < 0.001).

## DISCUSSION

This is the first report to directly compare the diagnostic performance of NBI, M‐NBI, PIT, and conventional EC diagnosis approaches with EC diagnosis with NP‐sign for LGA. In all lesions, EC with NP‐sign had a significantly higher accuracy, specificity, and PPV for predicting colorectal LGA compared with NBI, M‐NBI, PIT, and conventional EC; these findings were confirmed by calculating AUC.

Polyps ≤5 mm in size have an extremely low prevalence of invasive cancer and advanced histology.^2^ In one study, among resected‐and‐discarded lesions, no patients died from colorectal cancer related to the procedure, and there were no local and/or distant recurrences detected.^26^ Therefore, the “resect‐and‐discard” strategy has been proposed and is attracting attention because it reduces the effort and cost related to pathological diagnosis after polypectomy.^27^ However, a small number of colorectal carcinomas ≤5 mm in size has been confirmed.^6^ For example, one study reported that the average size of 10 lesions of intramucosal carcinoma without adenoma showing slight depression was 5.1 mm,^28^ and 2.0% of diminutive tumors were carcinoma, especially depressed‐type tumors, which had a significantly higher frequency of high‐grade dysplasia or submucosally invasive carcinoma.^29^ Furthermore, patients with villous component or high‐grade dysplasia have a high risk of future metachronous cancer.^11^, ^30^ Additionally, patients who underwent endoscopic resection of a high‐grade adenoma may be at high risk of developing further adenomas with high‐grade dysplasia or carcinoma.^31^, ^32^ An international survey of nine endoscopy societies worldwide revealed that one of the important reasons for the non‐implementation of the “resect‐and‐discard” strategy was the fear of making an incorrect endoscopic diagnosis of not only non‐neoplastic lesions but also LGA.^33^ This may be based on the fact that Western endoscopists usually use NBI without magnification and are less confident in their diagnosis. Therefore, it is critical for endoscopists to be able to diagnose LGA with high confidence to perform the “resect‐and‐discard” strategy in clinical practice.

M‐NBI and PIT diagnoses are currently used for the differential diagnosis of neoplastic lesions from non‐neoplastic lesions in the colon. The diagnostic accuracy of M‐NBI in distinguishing neoplastic from non‐neoplastic lesions was significantly better than that of non‐magnifying NBI.^9^ Regarding PIT diagnosis, one study reported that the diagnostic accuracy increases in the order of conventional view, chromoendoscopy, and chromoendoscopy with magnification.^10^ While magnifying endoscopic diagnostics (M‐NBI and PIT) are excellent modalities for diagnosing colorectal neoplastic lesions from non‐neoplastic ones, the ability to differentiate LGA from advanced lesions has not been established.

We previously showed that the NP‐sign in EC images is an indicator of colorectal LGA with high accuracy.^17^ Furthermore, the validation study for EC with NP‐sign showed a high concordance rate. In this study, EC with NP‐sign had a significantly higher ability for predicting LGA than NBI, M‐NBI, PIT, and conventional EC diagnosis in all lesions. However, the sensitivity and NPV of EC with NP‐sign were inferior to that of conventional EC; this was because the NP‐sign is not always found in LGAs. In lesions smaller than 5 mm, the diagnostic ability of EC with NP‐sign showed no significant differences from M‐NBI, PIT, and EC diagnosis, however, this could have been influenced by the rather small sample size of advanced lesions among these lesions (*N* = 22). Also, high diagnostic ability with four conventional modalities may reflect the fact that only experts performed the investigation in this study.

Only several images^1^, ^2^, ^3^ are required to diagnose NP‐sign for lesions less than 5 mm. There was no significant difference in the visibility of NP‐sign between the ultra‐high magnification of the prototypes and CF‐K290ECI. Double staining for EC observation does not mean spraying two agents separately, but it is a single procedure as we spray mixed solutions in one step. Western endoscopists who are not accustomed to using magnifying endoscopy may be reluctant to bring this method into daily practice. Nevertheless, since our data presents a strong advantage of EC by observing NP‐sign, it would be worthwhile to introduce the method even if it takes some time and effort. In Western countries, magnifying endoscopic observation is not widely used, and implementation of a “resect‐and‐discard” strategy is advocated among lesions less than 5 mm with high confidence level using non‐magnifying endoscopy.^3^, ^4^, ^5^, ^27^ We suggest that the NP‐sign with EC will contribute to the ‘resect‐and‐discard’ strategy regardless of lesion size since this method proved to be more accurate than other modalities in the present study.

Currently, a high adenoma detection rate and superior diagnostic ability for colorectal cancer with artificial intelligence (AI) have been reported.^34^, ^35^ In the future, it may be possible to automatically diagnose LGA with AI utilizing our present result.

The present study has some limitations. First, this was a single‐institutional investigation and a retrospectively designed study. Consequently, a regional or institutional bias could exist. In addition, whether endoscopists had low or high confidence was unclear. Second, EC observation was not performed for all neoplastic lesions in our facility. Furthermore, lesions diagnosed by non‐experts were not included. Consequently, a selection bias could exist. A prospective multicenter study including non‐experts should be performed, and the confidence level should be described.

In conclusion, we have reconfirmed that NP‐sign is an excellent indicator of colorectal LGA, showing additional diagnostic potential compared with conventional endoscopic modalities: EC diagnosis with NP‐sign showed a significantly higher specificity, PPV, accuracy, and AUC for predicting LGA compared with NBI, M‐NBI, PIT, and conventional EC diagnosis. Using EC diagnosis with NP‐sign will allow for the diagnosis of colorectal LGA more accurately in real time and help identify neoplastic lesions that can be managed by the “resect‐and‐discard” strategy, with higher confidence than before.

## CONFLICT OF INTEREST STATEMENT

None.
